# The Pan-American Medical Congress

**Published:** 1893-03

**Authors:** 


### NOTICES.

### The Pan-American Medical Congress.

The effort in behalf of the P. A. M. C., is even now showing in good results. The sections are all well organized, and promise good work in nearly every line. The Spanish speaking states have responded in many cases, and we may look for much of interest from them.

The object of the officers seems to be that of scientific attainments, and ample time for the work of the sections with no more time taken up in general session than is necessary for the transaction of the business which should come before it.

The Section on Oral and Dental Surgery is well organized, and a number of good papers promised from good men. The tendency of the papers seems to be more that of the surgical and anatomical part of dentistry rather than that of mechanical dentistry which seems fitting for a section of a medical congress.

The officers of this section do not look for great numbers, but feel assured of the success in scientific results, believing more good is done for the profession at large with one good practical or scientific paper, and ample time for its discussion, than many papers hurriedly read with little or no time for discussion.

The discussion of papers often brings out practical points and adds to their value which otherwise would be lost.

The publication of papers with their written and verbal discussion gives the whole profession the full value, and this seems to be the aim of the officers of this section.

